# Complex Aerogels Generated from Nano-Polysaccharides and Its Derivatives for Oil–Water Separation

**DOI:** 10.3390/polym11101593

**Published:** 2019-09-29

**Authors:** Hajo Yagoub, Liping Zhu, Mahmoud H. M. A. Shibraen, Ali A. Altam, Dafaalla M. D. Babiker, Songmiao Liang, Yan Jin, Shuguang Yang

**Affiliations:** 1 State Key Laboratory for Modification of Chemical Fibers and Polymer Materials, Center for Advanced Low-dimension Materials, College of Materials Science and Engineering, Donghua University, Shanghai 201620, China; hajoyagoub@hotmail.com (H.Y.); al-tam2013@hotmail.com (A.A.A.); dafaalla33034@outlook.com (D.M.D.B.); 2 Department of Textile Engineering, Faculty of Industries Engineering and Technology, University of Gazira, Wad-Madani, P.O. Box 20, Sudan; shibraen@yahoo.com; 3 R & D Center, Vontron Membrane Technology Co., Ltd., Guiyang 550000, China; liangsongmiao@crrcgc.cc (S.L.); jinyan.nt@crrcgc.cc (Y.J.)

**Keywords:** cellulose nanofiber, chitin nanocrystals, cationic guar gum, aerogel, oil absorption

## Abstract

The complex aerogel generated from nano-polysaccharides, chitin nanocrystals (ChiNC) and TEMPO-oxidized cellulose nanofibers (TCNF), and its derivative cationic guar gum (CGG) is successfully prepared via a facile freeze-drying method with glutaraldehyde (GA) as cross-linkers. The complexation of ChiNC, TCNF, and CGG is shown to be helpful in creating a porous structure in the three-dimensional aerogel, which creates within the aerogel with large pore volume and excellent compressive properties. The ChiNC/TCNF/CGG aerogel is then modified with methyltrichlorosilane (MTCS) to obtain superhydrophobicity/superoleophilicity and used for oil–water separation. The successful modification is demonstrated through FTIR, XPS, and surface wettability studies. A water contact angle of 155° on the aerogel surface and 150° on the surface of the inside part of aerogel are obtained for the MTCS-modified ChiNC/TCNF/CGG aerogel, resulting in its effective absorption of corn oil and organic solvents (toluene, n-hexane, and trichloromethane) from both beneath and at the surface of water with excellent absorption capacity (i.e., 21.9 g/g for trichloromethane). More importantly, the modified aerogel can be used to continuously separate oil from water with the assistance of a vacuum setup and maintains a high absorption capacity after being used for 10 cycles. The as-prepared superhydrophobic/superoleophilic ChiNC/TCNF/CGG aerogel can be used as a promising absorbent material for the removal of oil from aqueous media.

## 1. Introduction

Oily wastewater generated from different industrial processes, oil production, and transportation has become a worldwide problem as it creates ecological problems posing a great threat to the environment, human health, and national development [1]. Various methods have been developed for the separation of oil–water mixtures, including absorption [2], gas flotation [3], flocculation [4], ultrasonic techniques [5], and filtration [6]. Among these methods, the absorption method stands out due to its facile operability, significant capacity for oil capture, and high cost-efficiency [7,8,9]. Such sorbents are not only important on a large scale, such as in supertanker accidents, but also in chemical laboratories, production sites, and even households. An ideal absorbent material should be lightweight and have a porous structure that allows for the absorption of a great amount of oil, superhydrophobic/superoleophilic properties that ensure its high efficiency in the selective absorption of oil, high stability and reusability, and low fabrication and material cost [10]. Recently, much research has focused on the development of sustainable absorbent materials for oil–water separation based on natural products due to environmental concerns [11,12,13].

Polysaccharides, as natural macromolecular carbohydrate polymers, are considered as a renewable resource and offer a number of advantages. These advantages include biodegradability, biocompatibility, low processing costs, bioactivity, non-toxicity, and environmental friendliness [14,15,16,17]. More importantly, polysaccharides possess many reactive functional groups such as hydroxyl, amino, and carboxylic acid groups on their backbones that can readily be functionalized to yield a wide range of polysaccharide derivatives [15,17]. Cellulose and chitin are the most abundant polysaccharide polymers in the earth, which can be found in many different plants, some animals, fungi, and bacteria [18,19]. They are consisted of both crystalline and amorphous domains, though the amorphous region can be removed by using a controlled acid or enzymatic hydrolysis, or an oxidation reaction by using 2, 2, 6, 6-tetramethylpiperidine-1-oxyl (TEMPO) or nitric acid/sodium nitrite [20,21,22,23,24]. The released crystalline segments are usually referred to as nanocrystals, nanowhiskers, or nanofibers, and have been demonstrated to have various potential applications by many researchers [25,26,27,28], including their use in the preparation of aerogels for oil–water separation [29,30,31,32].

Aerogel, an intriguing and highly porous solid material prepared by replacing the liquid solvent in a gel with air while simultaneously keeping the three-dimensional (3D) network structure, has drawn significant attention as an efficient oil–water separation material due to its low density and large specific surface area [33,34,35]. Recently, aerogels based on carbon nanotubes and graphene have been applied to oil–water separation with very high sorption capacities and good recyclability [36,37,38,39]. However, the limitations of their manufacture are expensive apparatuses and complex technologies, which may restrict their practical applications. Therefore, researchers are making a great effort to explore new aerogel materials with excellent properties and low cost for oil–water separation [40,41]. One frontier directs to the assembly of the nanomaterials from natural resources, such as nano-polysaccharide and its derivatives. For example, Rafieian et al. fabricated a nanocellulose-based aerogel with low density and high porosity [42]. The surface of the aerogel was modified with hexadecyltrimethoxysilane to obtain hydrophobicity to absorb and remove oil and organic pollutants from water. Li et al. prepared cellulose nanofibers aerogels with controllable surface wettability by grafting poly (N, N-dimethylamino-2-ethyl methacrylate) (PDMAEMA) polymer brushes [43]. The surface of the resultant aerogel was hydrophobic in the ambient environment, but gradually changed from hydrophobic to hydrophilic in the presence of CO_2_. Therefore, the modified aerogels allowed for the on-off switching of the oil–water mixture separation process. Zhao et al. developed a composite sponge with chitin/halloysite nanotubes for oil–water separation [44]. After the introduction of lipophilic alkyls on the surface, the resultant sponge became hydrophobic and showed excellent absorption capability for various oils and organic solvents. The sponge also exhibited oil–water separation efficiency as high as 98.7%. Duan et al. synthesized a hydrophobic and oleophilic chitin sponge via a freeze-drying method followed by thermal chemical vapor deposition (CVD) of methyltrichlorosilane (MTCS) [45]. The sponges efficiently collected organic solvents on and under the surface of water.

We have previously reported a method to prepare complex membranes with porous structures by mixing oppositely charged polyelectrolytes generated from polysaccharide materials, which exhibited good performances in separating oil–water emulsions [46,47]. In this research, we further explore this method to fabricate porous complex aerogels using polysaccharide-based nanomaterials and study their absorption performances. The negatively charged cellulose nanofibers extracted from the bark of velvetleaf (Abutilon theophrasti) and the positively charged chitin nanocrystals from crab shells are prepared and used to form a complex gel solution with cationic guar gum and glutaraldehyde as a cross-linking agent. The complex gel solution is then turned into macroscopic aerogels by a freeze-drying method. The as-prepared complex aerogels are shown to have a highly porous structure. After a hydrophobic modification with MTCS through a CVD process, the modified aerogel exhibits a durable hydrophobicity and can be used to efficiently absorb oils and organic solvents beneath the surface or at the surface of water with excellent absorption performances and recyclability.

## 2. Experimental 

### 2.1. Materials

The bark of Abutilon theophrasti was provided by College of Textile, Gazira University, Wad-Madani, Sudan. Chitin (BioReagent), acetic acid (≥99.5%), glutaraldehyde (GA, 25.0–28.0% aq. soln.), and sodium bromide (≥99.0%) were bought from Sino Pharm Chemical Reagent Co. Ltd (Shanghai, China). Cationic guar gums (CGG) (the viscosity of 1.0 wt.% water solution is ~3000 mPa·s, DS = 1.2) was provided by Yan Cheng Xin Yuen Chemical Co. Ltd (YanCheng, China). 2, 2, 6, 6-tetramethylpiperidine-1-oxyl (TEMPO, 98%) was obtained from Aladdin Industrial (Shanghai, China). Hydrochloric acid (HCl, 36 wt.%) was purchased from Ping Hu Chemical Ltd (Pinghu, China). Potassium hydroxide (KOH, ≥85.0%) and sodium hydroxide (NaOH, ≥96.0%) were purchased from Shanghai Ling Feng Chemical Reagent Co. Ltd (Shanghai, China). Sodium chlorite (NaClO_2_, 80%) was supplied from Sigma-Aldrich (Shanghai, China). Sodium hypochlorite (NaClO, 11–14% available chlorine) was provided by Alfa Aesar (Shanghai, China) and methyltrichlorosilane (MTCS, 98%) was provided by Thermo Fisher Scientific (Shanghai, China). Sodium acetate (≥99.0%) was obtained from Shanghai Shisi Hewei chemical Industry Limited Company (Shanghai, China).

### 2.2. Preparation of TEMPO-Oxidized Cellulose Nanofibers (TCNFs) from the Bark of Abutilon Theophrasti 

**Figure 1:**
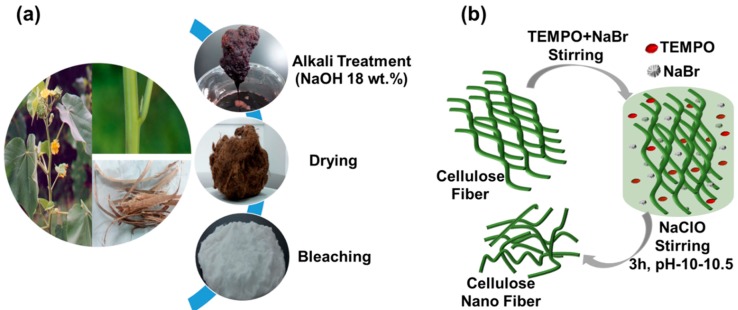
(**a**) Extraction cellulose fibers from the bark of Abutilon theophrasti and (**b**) preparation of 2, 2, 6, 6-tetramethylpiperidine-1-oxyl (TEMPO-oxidized cellulose nanofibers.

The cellulose fibers were first extracted from the bark of Abutilon theophrasti by an alkali treatment followed by bleaching [48,49], as shown in Figure 1a. Typically, 40 g of bark was treated with 600 mL sodium hydroxide solution with a concentration of 18 wt.% at 90 °C for 3 h. The resultant fibers were then washed thoroughly with water to remove the dissolved materials and were continually washed by water until the decanted solution had a neutral pH, then dried in the oven at 60 °C for 24 h (yield: 35.2%). A total of 12 g of the dried fibers were then bleached in a 500 mL of 0.3 M sodium acetate buffer, containing 8.5 g of NaClO_2_, for 6 h at 80 °C, and then thoroughly rinsed with DI water until neutral pH. The obtained cellulose fibers were then dried in an oven at 60 °C for 24 h (yield: 83.4%).

The bleached cellulose fibers were further treated following a TEMPO-mediated oxidation process [49,50], as shown in Figure 1b. A total of 5 g of the bleached fibers were suspended in water (500 mL) containing TEMPO (0.1 g) and sodium bromide (1.0 g). The TEMPO-mediated oxidation of the cellulose fibers was started by adding 11 wt.% NaClO solution (18.0 g). The mixed solution was continuously stirred at room temperature (RT) for approximately 3 h and the pH was maintained to be 10–10.5 by adding 2 wt.% NaOH until no more consumption of alkali was observed, indicating that the reaction was complete, the oxidation process was then stopped by adding 5 mL ethanol. The fibrous product was washed thoroughly with water with the help of centrifugation and sonication-assisted redistribution for three times and then stored in DI water at 4 °C until further use or characterization. The yield was determined to be approximately 73.1% by drying and weighing a small amount of the supernatant.

### 2.3. Preparation of Chitin Nanocrystals (ChiNC)

Chitin nanocrystals were prepared via acid hydrolysis [51]. The detailed preparation process of the ChiNC used in this research can be found in our previously published study [46]. The yield of resultant ChiNC was determined to be approximately 66%, and the final product was stored in DI water until further use or characterization.

### 2.4. Preparation of Complex Aerogels

**Figure 2:**
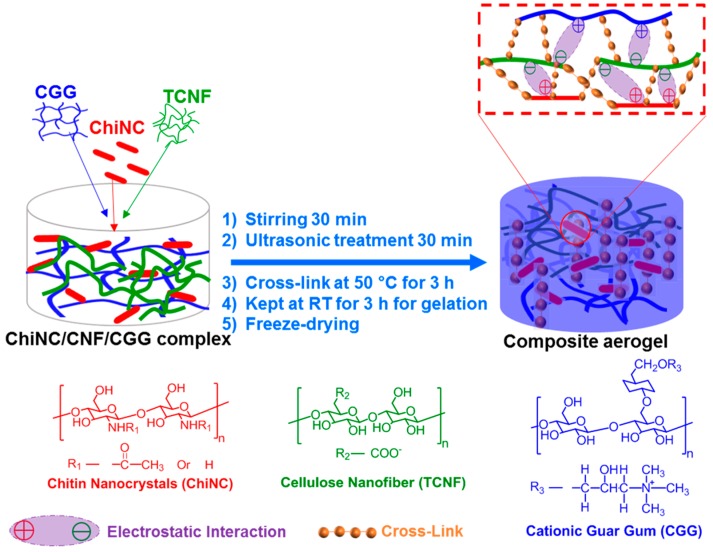
Schematic of preparation of the cross-linked chitin nanocrystals (ChiNC)/cellulose nanofibers (TCNF)/cationic guar gum (CGG) aerogel.

The ChiNC and TCNF suspensions, and the CGG solution, all with a concentration of 1.27 wt.% were prepared first. The complex solution was obtained by mixing 6 g of ChiNC suspension, 2 g of TCNF suspension, and 2 g of CGG solution under vigorous stirring for 30 min and ultrasonic treatment for 30 min. A total of 0.2 g of GA was then added to the mixed complex solution. The resulting mixture was continuously stirred at 50 °C for 3 h and then kept at RT for 3 h for gelation. Then the resultant hydrogel was frozen for 24 h at −18 °C, followed by freeze-drying for 24 h to remove all the water and obtain the complex aerogel. The procedure is illustrated in Figure 2.

### 2.5. Hydrophobic Modification of the Complex Aerogels

The modification of aerogels was performed by CVD of MTCS [45]. The aerogel was placed in a desiccator with a 25 mL beaker containing 1 mL of MTCS. The desiccator was sealed and kept at RT for 24 h. After treatment, the aerogel was kept in a vacuum oven at RT for 24 h to eliminate the excess saline and obtain the hydrophobic aerogel.

### 2.6. Absorption Performance Test

The obtained MTCS-modified aerogel was cut into small samples, each with a size of 5 mm × 5 mm × 10 mm, for the absorption tests. The samples were weighed before the test and immersed into 25 mL of oil (corn oil, n-hexane, toluene, and trichloromethane) for 5 min. After the immersion, the samples were removed from the oil, wiped with filter papers to remove the excess oil (or organic solvents) on the surface, then weighed. The absorption capacity (Q) was then calculated according to the following equation [52]:

(1) $$Q\;=\;\frac{W_{t\;}{-\;W}_{o}}{W_{o}},$$

where W_o_ is the weight of dry aerogel and W_t_ is the total weight of wet aerogel.

A self-made apparatus was used to carry out the continuous oil absorption experiments. One end of a tube was fixed at the center of the modified ChiNC/TCNF/CGG aerogel, while the other end of the tube was connected to a vacuum system. When the vacuum was switched on, the aerogel would continuously absorb oil from oil–water mixtures and pass the oil into the attached collector.

### 2.7. The Cycling of the Modified ChiNC/TCNF/CGG Aerogel

The cycling experiments of the modified ChiNC/TCNF/CGG aerogels were performed via absorption–distillation for organic solvents with a low boiling point (toluene, n-hexane, trichloromethane, etc.), and via a vacuum suction process for corn oil. For the absorption–distillation process, the organic solvent-absorbed aerogel was regenerated by heating at a temperature around the boiling point of the absorbed solvent in a venting hood to remove the absorbed organic solvents. Then the regenerated aerogel was used in the next absorption cycle. For the absorption-vacuum suction process, the corn oil absorbed by the aerogels was extracted by a vacuum suction process to regenerate the aerogels which were then used for the next cycle. In the cycling experiments, the absorption capacity of the modified ChiNC/TCNF/CGG aerogel was determined with the same method described previously.

### 2.8. Characterization

The Zeta potential and particle size distribution of the TCNF and ChiNC were measured using the Zetasizer Nano ZS (Malvern Panalytical, Worcestershire, UK), equipped with a 4 mV He-Ne laser operating at λ = 632.8 nm and the surface zeta potential was measured using the Surface Zeta Potential Cell (ZEN 1020) from Malvern. FTIR spectra of the samples were measured on an FTIR spectrometer (Nicolet 8700, Thermo Fisher Scientific, Waltham, MA USA) with a spectral resolution of 4 cm^−1^. A total of 32 scans were applied to obtain the spectra with an acceptable signal-to-noise ratio. X-ray photoelectron spectra (XPS) were obtained using an RBD upgraded PHI-5000C ESCA system (PerkinElmer, Waltham, MA, USA) with Al Kα radiation. Transmission electron microscopy (TEM) images were obtained from a multipurpose, 200 kV analytical electron microscope (JEM-2100, Joel, Tokyo, Japan). Scanning Electron Microscopy (SEM) images were obtained on the Hitachi SV-8010 electron microscopy (Hitachi, Tokyo, Japan). The Brunauer–Emmett–Teller (BET) method was used to determine the average pore diameter and the pore distribution. The samples were outgassed at 100 °C for 6 h under vacuum before running the nitrogen adsorption at 77 K with an ASAP 2020 accelerated surface area and porosimetry system (Micromeritics Instrument Corp, Norcross, GA, USA). The compression stress-strain curves of the aerogel were measured with an Instron Universal Testing system (Model 5966, Illinois Tool Works Inc., Glenview, IL, USA). The contact angle (CA) was performed with a contact angle measuring device (OCA20, Data Physics Instruments, Filderstadt, Germany). Thermogravimetric analysis (TGA) was performed on a TG 209-F1 Iris (NETZSCH, Upper Franconia, Germany) in N_2_ atmosphere with a heating rate of 10 °C/min.

## 3. Results and Discussion

### 3.1. Preparation and Characterization of TCNFs

Cellulose fiber was first extracted from velvetleaf, also known as Abutilon theophrasti or Chinese jute, and then TEMPO-oxidized to obtain TCNFs, in which the OHs at the C6 position were converted into carboxylic groups. Velvetleaf is grown in China for its bast fiber, which is used to make rope, cordage, bags, coarse cloth, fishing nets, paper stock, and for caulking boats [53]. The fiber obtained from the velvetleaf stems has a composition of 69% cellulose and 17% lignin, and has similar properties to that of ordinary bast fibers such as kenaf and hemp [54]. Velvetleaf fibers can be processed on the current kenaf processing machinery for textile, composite, automotive, and other fibrous applications [54]. However, velvetleaf is currently considered a weed and an agricultural problem in North America, which has initially been introduced to North America as a fiber crop from China. In an effort to find more applications for the velvetleaf and solving the agricultural problems at the same time, we have extracted TCNFs from Velvetleaf in this research. The yield was found to be comparable to that of the cellulose nanofibers extracted from jute fibers using a TEMPO oxidation method [49].

**Figure 3:**
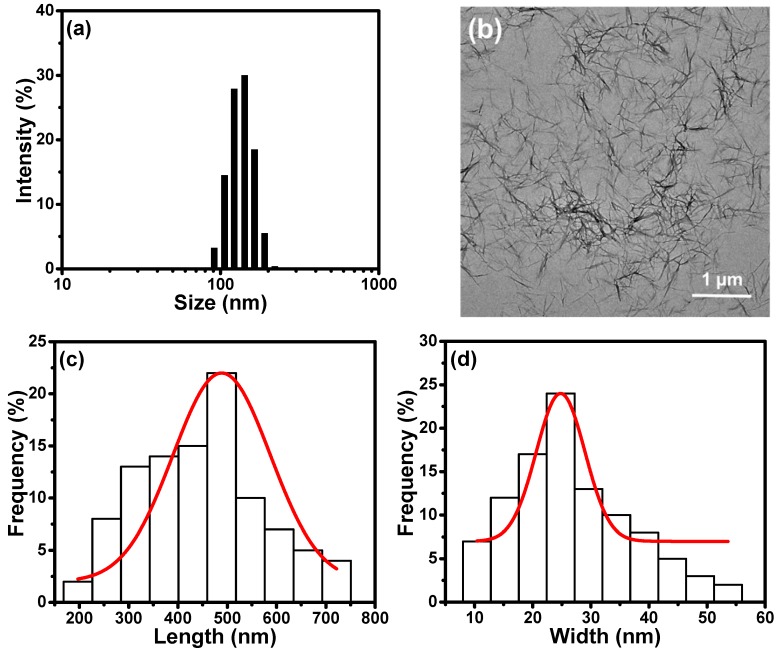
Characterization of the TCNFs: (**a**) The zeta size distribution, (**b**) TEM image, (**c**) length, and (**d**) width distribution of TCNFs.

Zeta potential, closely related to the surface charges of the nanoparticles, is measured by tracking the moving rate of the negatively or positively charges on the surface of nanoparticles across an electrical field. The zeta potential of TCNFs suspension was determined to be −37.7 mV, indicating the successful oxidation of hydroxyl groups in cellulose to be the negative carboxylate groups on the TCNF surfaces. According to the zeta size measurements, the average size of the TCNF particles is reported to be 450 nm with an excellent size distribution, as shown in Figure 3a. A TEM image is also shown in Figure 3b to provide a better understanding of dimension, shape, and size of TCNFs. Most of the TCNFs are randomly entangled, providing the web-like display in the image, while some of the individual fibers with the nano-sized lateral dimension are also observed. This suggests that the bleached cellulose fibers are successfully nanofibrillated through the TEMPO-oxidation. By measuring the sizes of 100 different nanofibers in the TEM images, the average length and width for TCNFs were determined to be 489 nm and 25 nm, respectively (Figure 3c,d).

**Figure 4:**
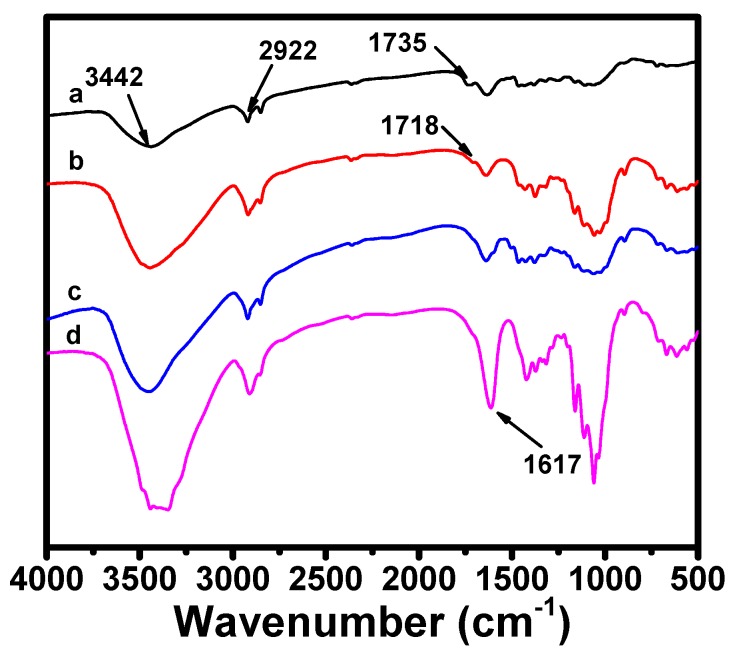
FTIR spectra of (**a**) velvetleaf bark, (**b**) alkali-treated velvetleaf bark, (**c**) bleached cellulose fibers, and (**d**) TCNFs.

The chemical structure changes of the velvetleaf bark, alkali-treated velvetleaf bark, bleached cellulose fiber, and cellulose nanofibers during TEMPO-oxidation were studied using FTIR. There are no significant differences observed in the FTIR spectra of the velvetleaf bark, alkali-treated velvetleaf bark, and bleached cellulose fibers (Figure 4). They all have a broad band around 3442 cm^−1^ assigned to –OH stretching, and peaks at 2922 cm^−1^ ascribed to C–H stretching. The peak at 1735 cm^−1^ in the velvet bark spectrum is attributed to the C=O stretching from the ketones and/or esters of hemicellulose, while this peak shifts to 1718 cm^−1^ after alkali treatment, and disappears in the bleached cellulose fiber and TCNF spectrum, which indicate that most of the hemicellulose in the velvet bark is dissolved and removed during the preparation of the TCNFs. However, TEMPO-oxidation leads to the appearance of a peak at 1617 cm^−1^, which is due to the stretching vibrations mode of the carboxylate groups from oxidation of the hydroxyl groups at the C6 position of the cellulose chain. Investigation on thermal stabilities of bleached fibers and TCNFs (Figure S1) also suggests the formation of sodium carboxylate groups at the C6 position of TCNFs as a significant decrease in the thermal degradation point is observed for TCNFs when comparing to the bleached before oxidation. This decrease is consistent with the thermal stability results reported by Isogai’s group [55].

### 3.2. Surface Morphology and Structure of the ChiNC/TCNF/CGG Aerogels

As was demonstrated in our previously reported study, mixing of polyelectrolytes with different charges is helpful in creating a complex membrane with porous structures [46,47]. In order to prepare the complex aerogel with a hierarchical porous structure, positively charged ChiNC was selected in this research to interact with the negatively charged TCNFs. The extensive existence of acetyl amino groups in chitin have proven to be helpful in the absorption of many organic hazardous pollutants [45,56]. Preparation of ChiNC was reported in our previous study through acid hydrolysis [46]. As discussed, due to the presence of some -NH_2_ groups that resulted from the partial deacetylation of chitin and the protonation of the amine groups in an acid environment, the prepared ChiNC was found to have a positively charged surface with a zeta potential of +52 mV when dispersed in a pH 3 solution. At the same time, the positively charged CGG, as a polysaccharide derivative and semi-flexible polymer, is introduced into the system as it is an excellent non-gelling thickener, as well as an effective volume and foam enhancer [57,58].

**Figure 5:**
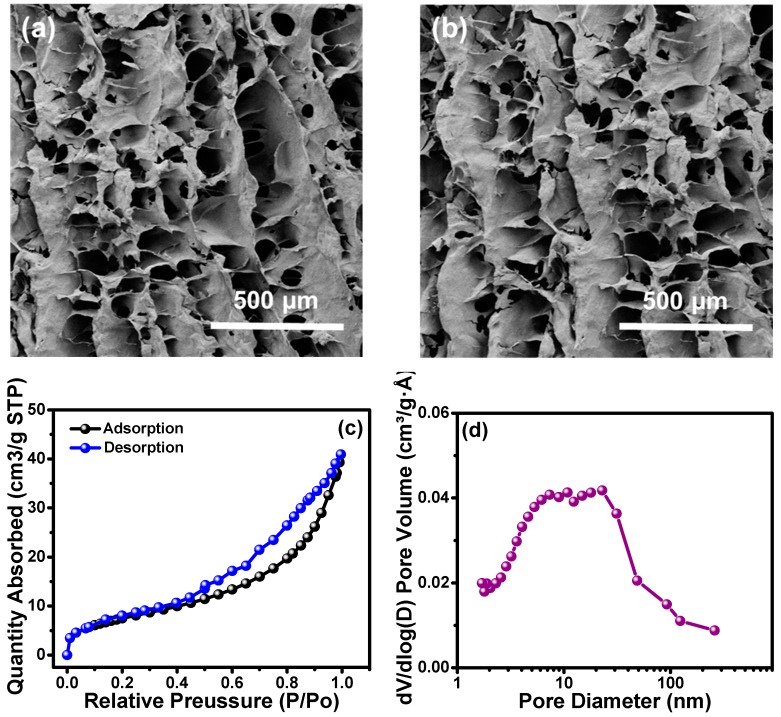
The SEM images of (**a**) unmodified and (**b**) modified aerogels; and the BET measurement of aerogels (**c**) adsorption and desorption, and (**d**) pore size.

The cross-linked ChiNC/TCNF/CGG aerogel was successfully prepared following a simple process consisting of mixing and freeze-drying, as shown in Figure 2. During mixing, the ChiNC, TCNF, and CGG interacted to form a complex, and the mixed solution gradually turned into a gel-like solution with a high viscosity. GA was added to help the formation of a chemically-linked network within the gel, which significantly improved the mechanical properties of the aerogel (Figure S2). A freeze-drying method was then used to remove the water in the system to obtain the dry aerogel. As shown in the SEM images (Figure 5a), a well-defined, interconnected, three-dimensional porous structure was observed for the dry ChiNC/TCNF/CGG aerogel. Due to the long aspect ratios and high surface area of TCNFs, they are very flexible and easily entangled, making them an excellent component in forming the pore walls in the aerogel. At the same time, the relatively stiff chitin chains can contribute support to the pore wall. Thus, the complexation of TCNF and ChiNC produced strong porous walls in the CGG matrix. The hydrophobic aerogel was fabricated by deposition of MTCS on the hydrophilic ChiNC/TCNF/CGG aerogels. As shown in Figure 5b, the highly porous structure was retained, confirming that the modification method was mild and would not change the original morphology of the resultant aerogel. The BET method was used to collect the nitrogen adsorption–desorption isotherms to further investigate the mesoporous structure within the ChiNC/TCNF/CGG aerogels, as shown in Figure 5c,d. Figure 5c shows that the isotherms exhibit a type IV curve as IUPAC classification, which suggests the existence of mesopores (pore size between 2 and 50 nm) and macropores (pore size larger than 50 nm) within the aerogel. The pore size distribution analysis (Figure 5d) indicates that the aerogel has a broad pore size distribution with sizes in the range of 2–100 nm, which are helpful in increasing the surface area of the aerogel.

**Figure 6:**
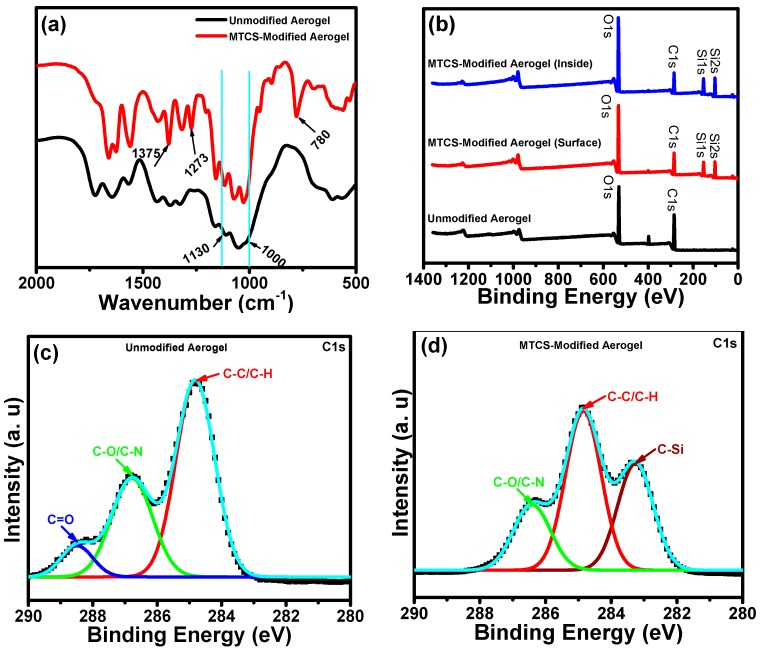
(**a**) FTIR spectra, (**b**) XPS survey spectra, (**c**) the high-resolution C1s spectrum of ChiNC/TCNF/CGG aerogels before modification, and (**d**) high-resolution C1s spectrum of ChiNC/TCNF/CGG aerogels after modification.

The successful salinization on the porous surface of the ChiNC/TCNF/CGG aerogels was confirmed by FTIR analysis of the unmodified aerogel and the modified aerogel (Figure 6a). After salinization using MTCS, the spectrum shows two obvious absorption bands at 781 cm^−1^ and 1273 cm^−1^, which correlates to the vibrations of the Si–O–Si and C–Si asymmetric stretching, respectively [45,59]. The strong peak at 1375 cm^−1^ is correlated to the bending of the -CH_3_ groups in MTCS. The characteristic peaks of the Si–O–Si bonds of siloxane compounds in the 1000–1130 cm^−1^ region overlap with that of the C–O bonds in the aerogel [60]. The FTIR result confirms the presence of strong interactions between the organosilane and the polysaccharide derivative aerogel, with covalent bonds between them, which is beneficial for the maintenance of hydrophobicity in practical application.

XPS analysis was also performed to monitor the surface structural changes of aerogels before and after modification. As shown in Figure 6b, the XPS spectra demonstrate that the original aerogel mainly contains carbon, oxygen, and nitrogen. While after modification, the XPS spectra of both the surface and inside of the aerogels show two new peaks at 154 eV and 103 eV, attributing to Si2s and Si2p, respectively, which indicate the presence of silicon within the aerogels [60]. Further, the high-resolution XPS spectra of C1s of ChiNC/TCNF/CGG aerogels before and after modification (Figure 6c,d) are provided to demonstrate the chemical structural changes of the aerogel. For the unmodified ChiNC/TCNF/CGG aerogels, the C 1s peak can be deconvoluted into three main peaks at 284.9 eV, 286.8 eV, and 288.4 eV, ascribing to C–C/C–H, C–O/C–N, and C=O, respectively [12]. While, after modification, the C1s peak is deconvoluted into three distinct peaks at 283.3 eV, 284.9 eV, and 286.4 eV, ascribing to C–Si, C–C/C-H, and C–O/C–N, respectively [61]. The appearance of the C–Si peak and the disappearance of C=O peak indicate the hydroxy groups on the surface of the aerogel have mostly reacted with MTCS and that the surface is covered with a thin layer of methylpolysiloxane which contains a large number of -Si–CH_3_ groups. Therefore, the surface wettability of the ChiNC/TCNF/CGG aerogel is expected to transform from hydrophilic to hydrophobic.

### 3.3. Surface Wettability and Absorption Performance of the ChiNC/TCNF/CGG Aerogel

**Figure 7:**
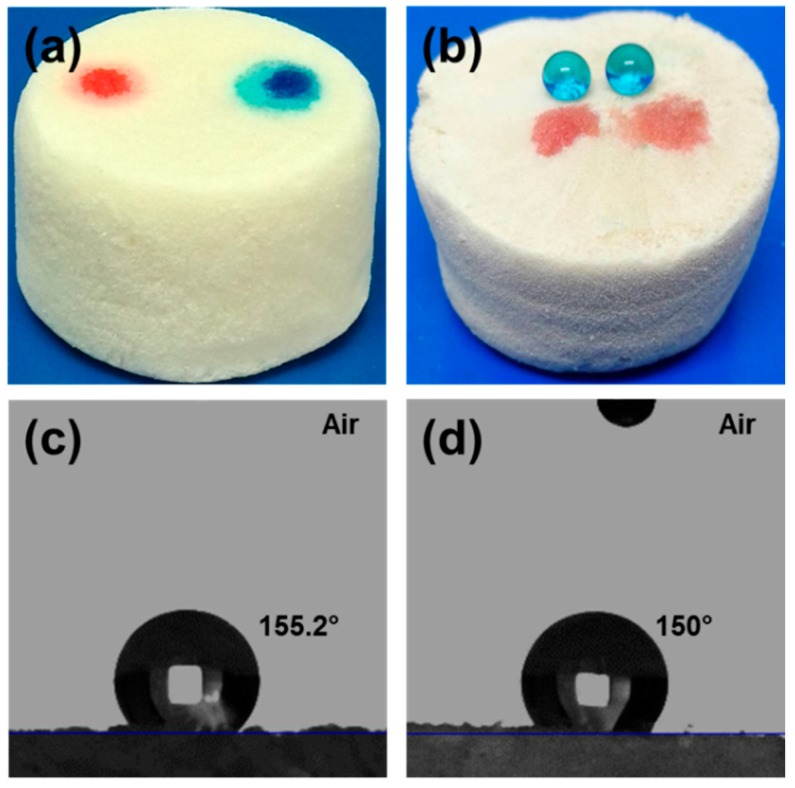
Photographs of water and oil droplets on the surface of the original aerogel (**a**) and the modified aerogel (**b**); the water contact angle of the modified aerogel: Water droplet is sitting on the outside surface (**c**) and inside surface (**d**) of the aerogel.

Figure 7a,b shows graphic pictures of the ChiNC/TCNF/CGG aerogel before and after saline modification. Morphology and the surface free energy are the two key factors to determine the surface wettability of materials [62,63]. The pristine ChiNC/TCNF/CGG aerogel is superhydrophilic due to the rough porous surface and the abundant hydroxyl groups presented in ChiNC, TCNF, and CGG, and thus it absorbs both water and oil quickly (Figure 7a). However, the MTCS-modification removes a large amount of the polar groups such as –OH groups in the aerogel and replaces them with hydrophobic organopolysiloxanes. Thus, the surface free energy of the aerogel is significantly decreased and the aerogel becomes superhydrophobic. In agreement with this assumption, water contact angles (WCA) are found to increase remarkably, and the water droplets can maintain the initial state as well as the round shape on the surface of the aerogel for a very long time. In contrast, the oil was instantly absorbed by the aerogel (Figure 7b). Figure 7c,d shows the WCA of the modified aerogel is approximately 155° on the aerogel surface and stays approximately 150° when tested on the surface of the inside part of the aerogel, which indicates that the whole modified aerogel is superhydrophobic, from surface to inside. In addition, the modified aerogel floats on the surface of water due to the hydrophobicity and the lightweight, while it absorbs oil and sinks inside the oil, as shown in Figure S3.

**Figure 8:**
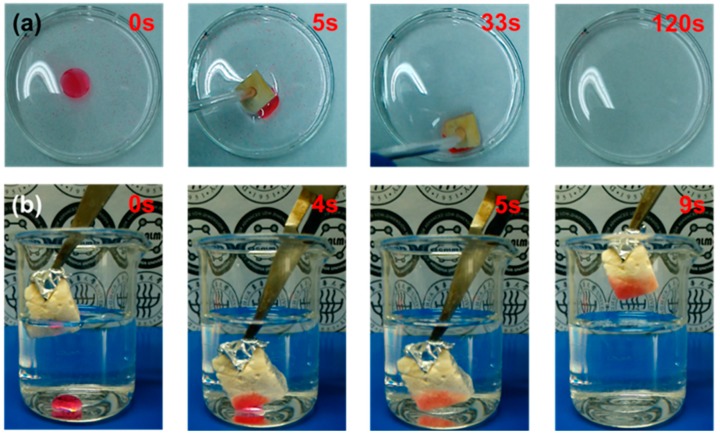
The absorption behavior of modified aerogels (**a**) from the surface of water, (**b**) from beneath the surface of water. Corn oil in (**a**) and trichloromethane in (**b**) were dyed with red oil dye for clarity.

**Figure 9:**
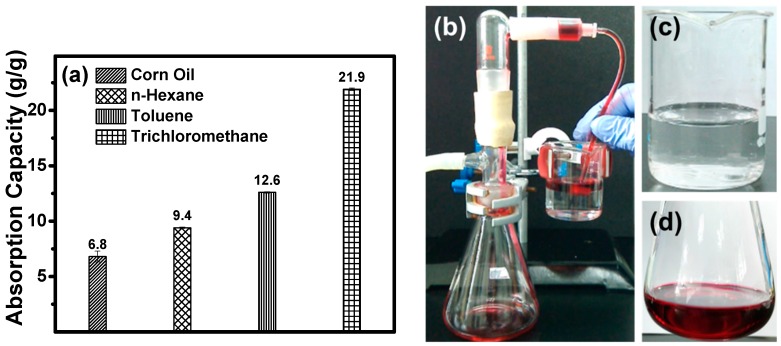
(**a**) The absorption capacities of aerogel for corn oil and organic solvents, (**b**) a self-made apparatus for continuous oil absorption, (**c**) water remained after the separation, and (**d**) the collected n-hexane (n-hexane was colored with red oil dye for clarity).

Due to their 3D porous structure and hydrophobicity, the modified ChiNC/TCNF/CGG aerogel shows a great potential for the facile removal of oil and organic solvents from the surface and beneath the surface of water. Figure 8a,b exhibits the absorption performance of the modified aerogels when they are contacted with a mixer of oil/organic solvent and water. The aerogel can rapidly absorb and remove corn oil, n-hexane, and toluene from the surface of the water (removing corn oil is shown as an example in Figure 8a), as well as the trichloromethane completely from beneath the surface of water as shown in Figure 8b. The absorption capacities of the modified aerogel for corn oil and organic solvents (toluene, n-hexane, and trichloromethane) were further investigated by a series of absorption experiments and determined using Equation (1). The results presented in Figure 9a suggest that the modified ChiNC/TCNF/CGG aerogel exhibits excellent absorption capacities for corn oil and organic liquids. In the absorption process, the liquids can rapidly penetrate into the interconnected 3D porous structure of the aerogels until an equilibrium is reached within a few minutes. The maximum absorption capacities (Q_m_) for corn oil, n-hexane, toluene, and trichloromethane were determined to be 6.8, 9.4, 12.6, and 21.9 g/g, respectively. The Q_m_ value is dependent on both the surface properties of the ChiNC/TCNF/CGG aerogel and features of the liquid (density, surface tension, hydrophobicity) [41,51]. The absorption capacity of the aerogel is also closely associated with the viscosity of the liquid because the viscosity significantly affects the movement velocity of liquids, which results in the different time needed to achieve the adsorption equilibrium [45]. Though the ChiNC/TCNF/CGG aerogels did not exhibit a significantly higher absorption capacity when compared with other natural materials-based aerogels (Table S1), we expected that the 3D porous structure of the aerogel could be further manipulated to increase the absorption surface area by controlling the amounts and ratio of the nano-polysaccharides, as well as the preparing conditions.

To recycle or reuse the separated oil, the absorbed corn oil or organic solvents must be collected or removed from the absorbents after absorption equilibrium. In this case, a self-made apparatus was designed and made in the lab for continuous oil–water separation experiments, which contained a vacuum set-up, a plastic tube, and the ChiNC/TCNF/CGG aerogels, as illustrated in Figure 9b. By using this system, the ChiNC/TCNF/CGG aerogel could quickly absorb n-hexane and repel water due to its superhydrophobic property, when the aerogel was placed at the interface of n-hexane (colored with oil red dye) and water. Once the vacuum pump was turned on, the n-hexane was continuously absorbed from the water surface into the ChiNC/TCNF/CGG aerogel and then removed from the aerogel through the tube. Simultaneously, a stream of n-hexane was formed in the plastic tube, and the amount of n-hexane in the mixture gradually decreased. Finally, the n-hexane layer was completely removed by using a small piece of the aerogel with the help of the vacuum pump (Movies S1, Supporting Information). No water droplets were observed in the collected n-hexane while no hexane was shown in the remaining water (Figure 9c,d).

### 3.4. Reusability of the Modified ChiNC/TCNF/CGG Aerogel

**Figure 10:**
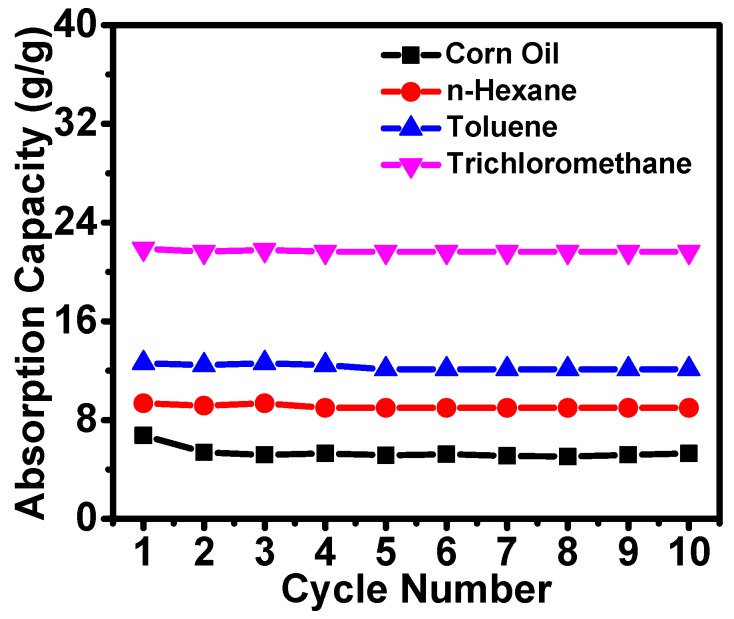
The cycling of the absorption capacity of the modified aerogels.

In practical applications, cycling and reusability are critical criteria for the absorbents. Hence, cycling experiments of the ChiNC/TCNF/CGG aerogel were performed. In order to regenerate the aerogels, the absorbed corn oil was removed from the aerogel by vacuum suction, while the absorbed organic solvents with relatively low boiling point (n-hexane, toluene, and trichloromethane) were removed by heating the aerogel at a temperature close to the boiling point of the solvents. The corn oil absorption-suction cycling experiments were performed using the device described previously, and it was found that the ChiNC/TCNF/CGG aerogel almost retained its original absorption capacity after 10 cycles. The recovery of corn oil was still near 100% after 10 cycles. The absorption of n-hexane, toluene, and trichloromethane was also tested by cycling experiments. Again, the absorption capacity of the ChiNC/TCNF/CGG aerogel remained unchanged after 10 cycles. No apparent deterioration of the aerogel was observed over 10 repetitions, and nearly 100% of the absorbed organic solvents could be removed in each cycle, as shown in Figure 10. These results demonstrate the excellent recyclability of the ChiNC/TCNF/CGG aerogel.

## 4. Conclusions

In summary, the porous ChiNC/TCNF/CGG aerogel was fabricated successfully via a simple freeze-drying method and then was treated by chemical vapor deposition of MTCS to obtain a hydrophobic aerogel. The deposition of MTCS was demonstrated to be effective in both the inside and at the surface of the aerogel and provided a stable hydrophobic property for the aerogel by forming a thin layer of methylpolysiloxane on the porous surfaces of the aerogel. The MTCS-modified ChiNC/TCNF/CGG aerogel showed great potential for the facile removal of oil and organic solvents from the surface and beneath the surface of water, due to their three-dimensional porous structure, hydrophobicity, and robust stability. With the assistance of vacuum suction, the MTCS-modified ChiNC/TCNF/CGG aerogel could continuously and effectively separate oil from oil–water mixtures and maintained a high absorption capacity after being used for many cycles. It was believed that such superhydrophobic ChiNC/TCNF/CGG aerogels could be used as a promising absorbent material for oil–water separation due to its facile preparation process and abundant materials resources. Furthermore, the idea of using the oppositely charged nano-polysaccharides to create the 3D aerogels with an interconnected porous structure was demonstrated to be effective, which can be further applied as a promising method to develop 3D porous systems with other charged nanomaterials. It is also recommended to pay attention to the formula and preparation conditions in such systems to generate porous absorbent materials with high surface areas. 

## Supplementary Materials

The following are available online at https://www.mdpi.com/2073-4360/11/10/1593/s1, Figure S1: TGA curves of bleached fibers and TCNFs, Figure S2: The compression test of (a) original aerogel and (b) cross-linked aerogel, Figure S3: The photographs of the oil absorption by MTCS-modified aerogel, and Table S1: Comparison of the absorption capacities of different absorption aerogels made of nature materials.
