# Supplementary material for: Complex Aerogels Generated from Nano-Polysaccharides and Its Derivatives for Oil–Water Separation
Source: Polymers (Basel). 2019 Sep 29;11(10):1593. doi: 10.3390/polym11101593 (PMC6835607; doi:10.3390/polym11101593)
Supplement: Supplementary file 1 [file polymers-11-01593-s001.zip › polymers-587592-SI/polymers-587592-supplementary.docx]

**Supporting Information**

**Complex Aerogels Generated from Nano-Polysaccharide and Its Derivatives for Oil Water Separation**

Hajo Yagoub^1,^ ^†^, Liping Zhu ^1, †,^ *, Mahmoud H. M. A. Shibraen ^2^, Ali A. Altam ^1^, Dafaalla M. D. Babiker ^1^, Songmiao Liang ^3^, Yan Jin ^3^ and Shuguang Yang ^1,^*

^1^ State Key Laboratory for Modification of Chemical Fibers and Polymer Materials, Center for Advanced Low-dimension Materials, College of Materials Science and Engineering, Donghua University, Shanghai 201620, China

^2^ Department of Textile Engineering, Faculty of Industries Engineering and Technology, University of Gazira, Wad-Madani, P. O. Box 20, Sudan

^3^ R & D Center, Vontron Membrane Technology Co., Ltd., Guiyang 550000, China

***** Correspondence: S. Yang (shgyang@dhu.edu.cn), L. Zhu (zhulp@dhu.edu.cn)

^†^ These authors contributed equally to this work.

**Figure S1.** TGA curves of the bleached fibers and TCNFs.

**Figure S2.** The compression test of (a) original aerogel and (b) cross-linked aerogel.

**
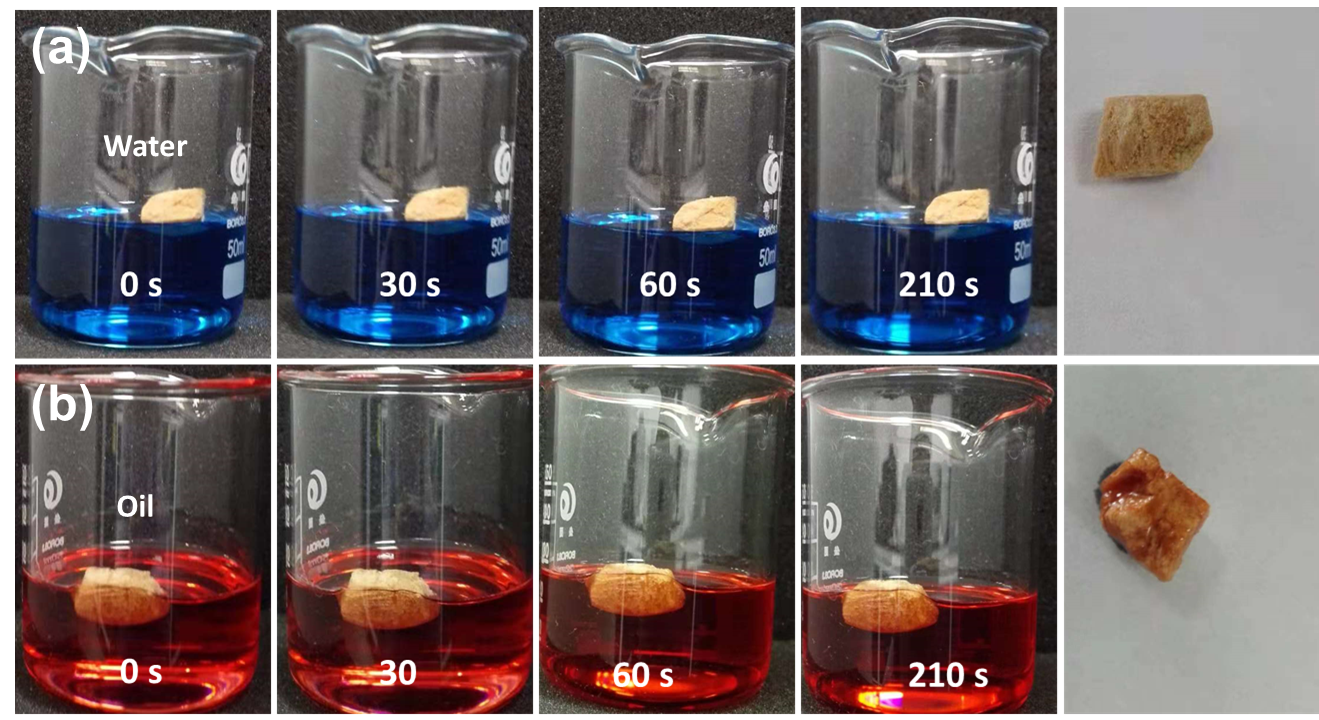
**

**Figure S3:** The photographs of the oil absorption by MTCS-modified aerogel.

Table 1. Comparison of the absorption capacities of different aerogels made of nature materials.

| **Absorbent Material** | **WCA (°)** | **Absorption Capacity (g/g)** | **Ref.** |
| --- | --- | --- | --- |
| Cellulose waste paper aerogel | 135.2 | 17.6–18 | [1] |
| Cellulose paper waste aerogel | 143–145 | 18.4–20.5 | [2] |
| Electrospun cellulose sponge | 141.2 | 15–37 | [3] |
| Cellulose aerogel | 141 | 30–59.3 | [4] |
| Bacterial cellulose aerogel | 146.5 | 80–185 | [5] |
| Cellulose/graphene aerogel | >150 | 80–197 | [6] |
| Nanocellulose sponge | 160 | 25–55 | [7] |
| Nanocellulose aerogel | >90 | 78.8–162.4 | [8] |
| Cellulose nanofiber aerogel | 133.5 | 16.8–17.8 | [9] |
| Chitin sponge | 145–148 | 29–58 | [10] |
| Chitosan/Cellulose aerogel | 152.8 | 13.77–28.20 | [11] |
| Chitin/halloysite nanotubes sponge | 88–98 | 3.9–11.2 | [12] |
| ChiNC/TCNF/CGG aerogel | 155 | 6.8-21.9 | This work |

**Reference**

1. Nguyen, S.T.; Feng, J.; Ng, S.K.; Wong, J.P.W.; Tan, V.B.C.; Duong, H.M. Advanced thermal insulation and absorption properties of recycled cellulose aerogels. *Colloids Surf. A: Physicochem. Eng. Aspects* **2014**, *445*, 128-134.
2. Nguyen, S.T.; Feng, J.; Le, N.T.; Le, A.T.T.; Hoang, N.; Tan V.B.C.; Doung, H.M. Cellulose Aerogel from Paper Waste for Crude Oil Spill Cleaning. *Ind. Eng. Chem. Res.* **2013**, *52*, 18386-18391.
3. Xu, T.; Wang, Z.; Ding, Y.C.; Xu, W.H.; Wu, W.D.; Zhu, Z.T.; Fong, H. Ultralight electrospun cellulose sponge with super-high capacity on absorption of organic compounds. *Carbohydr. Polym.* **2018**, *179*, 164-172.
4. Liao, Q.; Su, X.; Zhu, W.; Hua, W.; Qian, Z.; Liu, L.; Yao, J. Flexible and durable cellulose aerogels for highly effective oil/water separation. *RSC Adv.* **2016**, *6*, 63773-63781.
5. Sai, H.; Fu, R.; Xing, L.; Xiang, J.; Li, Z.; Li, F.; Zhang, T. Surface Modification of Bacterial Cellulose Aerogels’ Web-like Skeleton for Oil/Water Separation. *ACS Appl. Mater. Interfaces* **2015**, *7*, 7373-7381.
6. Mi, H.-Y.; Jing, X.; Politowicz, A.L.; Chen, E.; Huang, H-X.; Turng, L-S. Highly compressible ultra-light anisotropic cellulose/graphene aerogel fabricated by bidirectional freeze drying for selective oil absorption. *Carbon* **2018**, *132*, 199-209.
7. Phanthong, P.; Reubroycharoen, P.; Kongparakul, S.; Samart, C.; Wang, Z.; Hao, X.; Abudula, A.; Guan, G. Fabrication and evaluation of nanocellulose sponge for oil/water separation. *Carbohydr. Polym.* **2018**, *190*, 184-189.
8. Rafieian, F.; Hosseini, M.; Jonoobi, M.; Yu, Q.L. Development of hydrophobic nanocellulose-based aerogel via chemical vapor deposition for oil separation for water treatment. *Cellulose* **2018**, *25*, 4695-4710.
9. Zanini, M.; Lavoratti, A.; Lazzari, L.K.; Galiotto, D.; Pagnocelli, M.; Baldasso, C.; Zattera, A.J. Producing aerogels from silanized cellulose nanofiber suspension. *Cellulose*, **2017**, *24*, 769-779.
10. Duan, B.; Gao, H.; He, M.; Zhang, L. Hydrophobic modification on surface of chitin sponges for highly effective separation of oil. *ACS Appl. Mater. Interfaces* **2014**, *6*, 19933-19942.
11. Li, Z.; Shao, L.; Hu, W.; Zheng, T.; Lu, L.; Cao, Y.; Chen, Y. Excellent reusable chitosan/cellulose aerogel as an oil and organic solvent absorbent. *Carbohydr. Polym.* **2018**, *191*, 183-190.
12. Zhao, X.; Luo, Y.; Tan, P.; Liu, M.; Zhou, C. Hydrophobically modified chitin/halloysite nanotubes composite sponges for high efficiency oil-water separation. *Int. J. Biol. Macromol.* **2019**, *132*, 406-415.
